# Binge Eating Disorder: What Is the Role of Physical Activity Associated with Dietary and Psychological Treatment?

**DOI:** 10.3390/nu12123622

**Published:** 2020-11-25

**Authors:** Letizia Galasso, Angela Montaruli, Konrad S. Jankowski, Eleonora Bruno, Lucia Castelli, Antonino Mulè, Mirella Chiorazzo, Alberto Ricceri, Stefano Erzegovesi, Andrea Caumo, Eliana Roveda, Fabio Esposito

**Affiliations:** 1 Department of Biomedical Sciences for Health, University of Milan, Via G. Colombo 71, 20133 Milan, Italy; angela.montaruli@unimi.it (A.M.); lucia.castelli@unimi.it (L.C.); antonino.mule1@unimi.it (A.M.); andrea.caumo@unimi.it (A.C.); eliana.roveda@unimi.it (E.R.); fabio.esposito@unimi.it (F.E.); 2 IRCCS Istituto Ortopedico Galeazzi, Via R. Galeazzi 4, 20161 Milan, Italy; 3 Faculty of Psychology, University of Warsaw, Stawki 5/7, 00-183 Warsaw, Poland; kjankows@psych.uw.edu.pl; 4 Department of Research, Fondazione IRCCS Istituto Nazionale dei Tumori di Milano, Via G. Venezian 1, 20133 Milan, Italy; eleonora.bruno@istitutotumori.mi.it; 5 Department of Clinical Neurosciences, IRCCS San Raffaele Turro, Via S. d’Ancona 20, 20127 Milan, Italy; mirella.chiorazzo@gmail.com (M.C.); albertoricceri@hotmail.it (A.R.); erzegovesi.stefano@hsr.it (S.E.)

**Keywords:** binge eating disorder, physical activity, eating disorder symptoms, cognitive-behavioral therapy, dietary program, women

## Abstract

Binge eating patients present lower physical activity levels, which could be associated with lower exercise capacity. Specific physical activity can ensure broad beneficial results relating to eating disorders, depression, and body mass index (BMI) in bulimia; however, research on binge eating disorder (BED) is scarce. Our study aimed to investigate the effects of specific training as an addition to conventional treatment of eating disorder symptoms, anthropometric characteristics, and physical performance. Nineteen women with BED were included in a dietary and cognitive-behavioral therapy program. After medical examination, 10 women carried out Combined Aerobic and Anaerobic Exercise Training in addition to conventional treatment (CAAET group), whereas the remaining 9 followed the conventional treatment alone (CTRL group). All of the measurements were assessed before and after six months of treatment. In both groups, we observed a significant decrease in binge episodes, weight, and body mass index, and an increase in exercise capacity. Moreover, the CAAET group presented a greater improvement in aerobic performance than that observed in the CTRL group. Our results suggest that both interventions similarly improved BED symptoms. The addition of physical activity could be important in the long-term maintenance of both weight loss and reduction in binge episodes in BED patients.

## 1. Introduction

Binge eating disorder (BED) has an independent diagnosis and is characterized by frequent and persistent episodes of binge eating, accompanied by a loss of control and marked distress in the absence of regular compensatory behaviors that are characteristic of bulimia [1]. The binge episodes take place quickly, without the physical necessity to eat, and alone, due to the individual’s feeling of shame regarding their own behavior.

These episodes are associated with feeling depressed, disgusted, or guilty after overeating [1,2]. BED presents a higher prevalence in women than in men (3.5% and 2%, respectively), and underlying mechanisms may be different in both genders. Specifically, gender differences in eating behaviors depend on psychological and physiological differences driven by hormones, culture norms, and mechanisms of mood regulation [3,4], warranting separate analysis of BED in men and women.

Although obesity is not a criterion for BED diagnosis, a strong positive association exists between weight and BED [5]. Patients with BED and obesity display more eating disorder symptoms, greater increase in body mass, increased body dissatisfaction, lower self-esteem, and an altered quality of life compared to obese non-binge eaters [6]. BED patients are more likely to develop mood disorders, including depression and anxiety, and for these reasons, BED has been considered one of the most difficult psychiatric conditions to treat [7]. Cognitive-behavioral therapy (CBT) is the traditional BED treatment, designed to identify and challenge maladaptive cognitions of eating and body mass. CBT focuses primarily on stopping binges and not on weight loss [8].

In BED patients, both obesity and inactivity reduce physical activity levels [9,10]. Patients with BED and obesity are also more sedentary than age- and body mass-matched obese patients alone [11].

Aerobic exercises and yoga have been proven to reduce body mass index (BMI) and binge episodes [12,13]. Furthermore, physical activity combined with CBT is more efficient in reducing BED symptoms than CBT alone in bulimia [12]. Aerobic and strength training are generally used to trigger health benefits and to increase physical activity levels [14,15,16]. Evidence suggests that a combination of strength and aerobic training yields similar physiologic improvements as aerobic or strength training alone, without interference between them [14].

Existing studies on physical activity as an add-on to CBT in BED patients are scarce [6,13], but results from studies on bulimia are encouraging [12]. Importantly, those analyzing BED patients have limitations, such as relying on subjectively assessed physical activity (as opposed to objective indicators) and no control group [6], or assessing only physical symptoms rather than eating disorder symptoms [13].

Therefore, the aim of the current study was to address these limitations by investigating, in women with BED, the effects of structured Combined Aerobic and Anaerobic Exercise Training (CAAET) as an additional treatment to a dietary and CBT program on eating disorder symptoms, anthropometric characteristics, aerobic capacity, and muscle strength.

## 2. Materials and Methods

### 2.1. Participants

The study aim and procedure were explained to 65 eligible participants who gave their written informed consent before enrollment in the study. From the first group of 65 women with BED, 41 (63%) were discarded because of exclusion criteria, both for medical reasons (*n* = 31, 76%) and because they were unable to take part in exercise training (*n* = 10, 24%). Subsequently, of the remaining 24 women with BED, 4 voluntarily declined to participate (17%).

**Figure 1:**
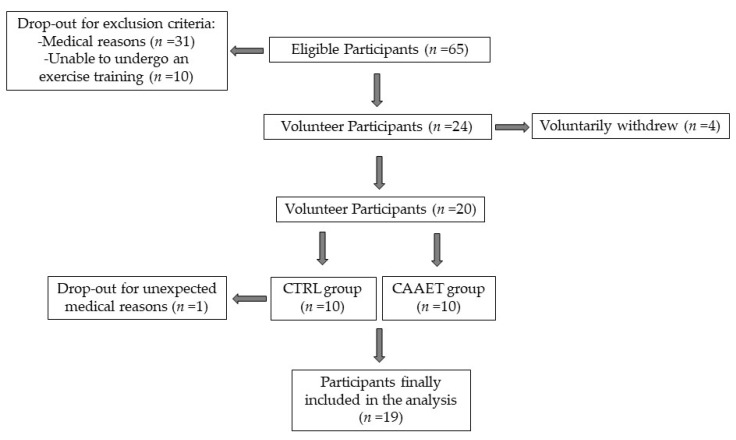
Participant recruitment. Study design and the participants’ adherence and dropout. CTRL, control group; CAAET, Combined Aerobic and Anaerobic Exercise Training (intervention group).

The study sample involved 20 women under treatment at the IRCCS San Raffaele Turro in Milan, Italy, with BED diagnosis using the Diagnostic and Statistical Manual of Mental Disorders, Fifth Edition (DSM-5) [1] and the Structured Clinical Interview (SCID-I) [17]. Ten women (50%) carried out a dietary and CBT program alone (CTRL group; mean age (±SD) 53 ± 13 years; mean height (±SD) 168 ± 9 cm) and 10 (50%) carried out a dietary and CBT program in combination with the CAAET (CAAET group; mean age (±SD) 54 ± 11 years; mean height (±SD) 162 ± 6 cm). Unfortunately, 1 woman from the CTRL group had to leave the experimental protocol for unexpected medical reasons before the beginning of the study, resulting in a final study sample of 19 women: 9 for the CTRL group (47%) and 10 for the CAAET group (53%) (Figure 1).

Inclusion criteria were BED diagnosis, age 18–75 years, body mass index (BMI) ≥ 30 kg/m^2^, and ability to undergo an exercise training after a medical examination.

Exclusion criteria were pregnancy, nursing, presence of a severe current psychiatric condition that required hospitalization in addition to the weekly clinical BED program, and a diagnosis of genetic obesity based on analysis of family history by a physician [18].

### 2.2. Ethics

BED women were invited to participate voluntarily in the study. A written informed consent was obtained before enrollment in the study, which was performed in line with the rules of the Declaration of Helsinki of 1975, revised in 2013. Approval was granted by the Ethics Committee of the San Raffaele Hospital, Protocol Number: TRDCA-01 (23 September 2014).

### 2.3. Experimental Design

During the study, all women maintained their individual multidisciplinary therapy, consisting of CBT and dietary program administered in outpatient care, from 8:00 a.m. to 5:00 p.m., Monday to Friday, over a period of 6 months.

Anthropometric values, eating disorder symptoms, and exercise capacity were assessed at baseline (PRE) and after 6 months of intervention (POST) in both the CTRL and CAAET groups. PRE was conducted during two visits. During the first visit, anthropometric and eating disorder symptom evaluations were conducted using questionnaires: Binge Eating Scale (BES) [19] and Bulimic Investigatory Test Edinburgh (BITE) [20]. During the second visit, patients performed the Six-Minute Walk Test (6MWT) and Squat Test (ST) on two different days, in random order, with 48 h of rest in between.

At POST, patients were evaluated again with the same procedures.

### 2.4. Experimental Procedures

#### 2.4.1. Anthropometric Measurement

Height and body weight were measured in triplicate without shoes and heavy clothes. Body mass index (BMI) was calculated as body weight in kilograms divided by the square of the height in meters (kg/m^2^).

#### 2.4.2. Eating Disorder Symptoms Evaluation

The Binge Eating Scale (BES) is a psychometric test to evaluate eating behavior, including emotional and cognitive symptoms in obese individuals. BES consists of 16 items with 4 response options. Responses are scored from 0 to 3 points and the total score is between 0 and 46: a score ≥17 indicates the possibility of BED [19].

The Bulimic Investigatory Test Edinburgh (BITE) is a validated questionnaire that identifies compulsive eating behaviors and reveals possible compensatory behaviors (vomiting, abstinence from food, use of laxatives). BITE consists of 33 items divided into two scales: the Symptom Scale, which detects all kind of symptoms, and the Severity Scale, which measures the intensity of the symptoms. In the Symptom Scale, a score ≥20 indicates the possibility of suffering from BED or Bulimia Nervosa; a score between 10 and 19 indicates a patient’s eating behavior is unusual but not pathological; a score under 10 indicates that there are no eating problems. In the Severity Scale, a score ≥10 indicates a high severity of bulimic behaviors; a score ≥5 suggests presence of bulimic behaviors [20].

#### 2.4.3. Exercise Capacity Assessment

The Six-Minute Walk Test (6MWT) has been shown to be a reliable and valid test to assess physical fitness in obese patients [21,22]. The 6MWT was performed outdoors, following the recommendations by the American Thoracic Society Statement [23]. At the beginning of the 6MWT, participants were asked to inform the trainers about pathological conditions that could interfere with physical performance.

For a period of 6 min, participants walked for 30 m as fast as they could without running or jogging.

The Squat Test (ST) was adopted to measure legs’ strength. Patients bent and extended their legs from a standing starting position with arms folded across the chest and their feet 20 cm apart. The objective was to reach a knee flexion of 90°. The score was the number of times that the participant could rise from a “seated position” to a full stand within 30 s [24,25,26].

### 2.5. Interventions

#### 2.5.1. Combined Aerobic and Anaerobic Exercise Training (CAAET)

The CAAET group attended the exercise program for 6 months, under the supervision of 4 sport therapists, including four weekly sessions of a total of 90 min: 60 min of aerobic activity, such as brisk walking, and 20 min of exercises for muscle strength. Each training session included 10 min of cool-down via static stretching at the end of the activity. All training sessions were supervised by the same trainers during the 6-month period.

#### 2.5.2. Cognitive-Behavioral Therapy (CBT)

The aim of CBT was to normalize the patient’s inappropriate eating behavior. It was performed by psychologists and each group session consisted of meetings of 90 min, three times per week [8,27].

#### 2.5.3. Dietary Program

The participants carried out a dietary program prescribed by a nutritionist. The lunches held during the week took place at the hospital under the supervision of the nutritionist, following the guidelines of the Mediterranean Diet. In addition, twice per week, the participants attended cooking classes to learn how to cook in a tasty and healthy manner. Furthermore, the participants were also invited to follow the dietary program at home.

### 2.6. Sample Size Calculation

For each group, we carried out a power analysis that focused on the ability of the study to detect changes in BES, the most clinically meaningful parameter of eating disorders. To perform the power analysis, we needed an estimation of the variability about Δ of BES. In our previous data (unpublished), subjects undergoing the BED traditional treatment showed a mean value of Δ equal to 8, SD = 3, and thus we set SD = 3. We decided that the magnitude of the clinical difference of interest in BES between the two groups was 5. By setting the power (1 − β) to 0.80 and the significance level α to 0.05 (2-tailed), we found that the sample size for each group must be approximately 7 subjects.

Calculations were carried out by assuming a Gaussian distribution of the values and equal variances between the groups (i.e., making reference to the pooled *t*-test). The power analysis was performed using the sample size calculator developed by Russel Lenth and available at http://www.stat.uiowa.edu/~rlenth/Power [28]. In the event of significant departure of the data from the Gaussian distribution (thus leading to the use of a non-parametric test to establish the difference between the two groups), we increased the sample size calculated for the pooled *t*-test by 15% [29]. Finally, we arrived at a sample size of 9 subjects for the CTRL group and 10 subjects for the CAAET group.

### 2.7. Statistical Analysis

SPSS 20 (IBM Corp. Released 2011. IBM SPSS Statistics for Windows, Version 20.0. Armonk, NY, USA: IBM Corp.), and R statistics software (Version 3.6.0) were used for analyses.

A normality check was conducted using the Shapiro–Wilk’s test. The analysis was conducted using parametric and non-parametric tests, due to small violations of the normality assumption. Because analysis of variance (ANOVA) is robust to small violations of the normality assumption [30], we decided to report the results obtained by the parametric tests (results of non-parametric tests were the same). Two-way ANOVA with repeated measures was used to investigate effects of time (PRE vs. POST) and group (CTRL vs. CAAET) on the following dependent variables: anthropometric characteristics (BMI and weight); eating disorder symptoms evaluation (BES, BITE Symptom, and BITE Severity); exercise capacity (6MWT and Squat Test). In the case of statistically significant interaction time × group, ANOVA was followed by Tukey-Kramer HSD post hoc tests. Finally, Cohen’s *d* was used to measure effect sizes and was calculated by dividing the difference between mean Δ scores by the average pooled standard deviation of the Δ. Effect sizes were interpreted according to the criteria supplied by Cohen [31] (*d* = 0.2 small; *d* = 0.5 medium; *d* = 0.8 large). The level of significance was set at α < 0.05. In both groups, anthropometric, eating disorder symptoms, and exercise capacity levels were expressed as mean values (±SD).

## 3. Results

Nineteen women were divided into CAAET (*n* = 10), or CTRL groups (*n* = 9) after medical examination. In the PRE condition, the two groups were homogeneous without any significant differences for all variables.

### 3.1. Anthropometric Measurement

Body weight and BMI were assessed both in the CAAET and in CTRL groups at PRE and POST conditions. The results showed that the CAAET and CTRL groups did not differ in body weight and BMI (Table 1), either at PRE or POST. In both groups, however, a significant reduction in body weight and BMI was observed after 6 months.

### 3.2. Eating Disorder Symptoms Evaluation

BITE Symptoms and Severity, and BES score, were assessed both in the CAAET and in CTRL groups at PRE and POST conditions. The results showed that the CAAET and CTRL groups did not differ in BITE Symptoms/Severity and BES (Table 2), either at PRE or POST. In both groups, however, a significant reduction in eating disorder symptoms appeared after 6 months.

### 3.3. Exercise Capacity Assessment

6MWT and ST were assessed both in CAAET and CTRL groups at PRE and POST conditions.

The results show that the CAAET and CTRL groups did not differ in 6MWT and ST (Table 3) at PRE. In both groups, a significant improvement in both 6MWT and ST was observed after 6 months. Furthermore, at POST, performance in 6MWT was greater in CAAET than in CTRL, indicating that patients in CAAET improved their performance in the walking test more than patients in the CTRL group.

### 3.4. Effect Sizes

Effect sizes (Cohen’s *d*) of differences between PRE and POST in the CAAET group in BMI (*d* = −0.9), BES (*d* = −1.2), BITE Severity (*d* = −0.9), and 6MWT (*d* = 1), were large. Effect sizes of differences between PRE and POST in the CAAET group in BITE Symptom (*d* = −0.7), body weight (*d* = −0.7), and ST (*d* = 0.7) were medium.

Effect sizes of differences between PRE and POST in the CTRL group in BES (*d* = −1) and BITE Symptom (*d* = −1.1) were large. Effect sizes of differences between PRE and POST in the CTRL group in 6MWT (*d* = 0.6) and BITE Severity (*d* = −0.6) were medium. Effect sizes of differences between PRE and POST in the CTRL group in BMI (*d* = −0.2), body weight (*d* = −0.2), and ST (*d* = 0.3) were small.

Finally, effect sizes of differences between CAAET and CTRL at POST in 6MWT (*d* = 0.9), BITE Severity (*d* = −0.8), and ST (*d* = 0.8) were large. Effect sizes of differences between CAAET and CTRL at POST in BMI (*d* = −0.5), BES (*d* = −0.6), BITE Symptom (*d* = −0.6), and body weight (*d* = −0.6) were medium.

## 4. Discussion

Our results suggest that women in both intervention groups achieved major improvements in anthropometric measures, eating disorder symptoms, and exercise capacity, and both interventions similarly improved BED symptoms. Furthermore, a greater improvement in aerobic performance was observed in the CAAET group compared to women in the CTRL group.

### 4.1. Anthropometric Changes after CAAET

Regarding anthropometric parameters, a significant reduction in body weight and BMI was observed in CAAET and CTRL women. These results mean that the two interventions had similar, positive effects in reducing body weight and BMI. However, the effect sizes of difference between PRE and POST in CAAET compared to the CTRL group showed a greater improvement for the women who followed the physical activity program (*d* body weight: −0.7 vs. −0.2, CAAET and CTRL, respectively; *d* BMI: −0.9 vs. −0.2, CAAET and CTRL, respectively). Physical consequences of BED are due to a comorbidity of obesity and sedentary lifestyle. Physical activity is associated with maintenance of weight loss over time in BED patients and with a reduction in binge eating episodes [32]. Our study is consistent with the scientific literature in which weight loss predisposes BED women to a higher level of physical activity.

### 4.2. Eating Disorder Symptoms Response to CAAET

After 6 months of intervention, both groups showed improvements in eating disorder symptoms, obtaining lower scores in all questionnaires. These results mean that the two interventions had similar, positive effects in reducing eating disorder symptoms of BED. As seen for the anthropometric values, with the exception of BITE Symptom, the effect sizes of differences between PRE and POST in CAAET compared to the CTRL group showed a greater improvement for the women who followed the physical activity program (*d* BES: −1.2 vs. −1, CAAET and CTRL, respectively; *d* BITE Severity: −0.9 vs. −0.6, CAAET and CTRL, respectively). It is known that abstinence from eating episodes is an important goal in the treatment of binge eating problems [33].

Furthermore, the combination of diet and exercise in conjunction with CBT is likely more beneficial for weight loss, constituting the best way to ameliorate psychological state: depression, anxiety, and eating disorder scores show an important decrease compared to results in people who follow only the CBT or the CBT with the addition of a nutritional program [34]. In agreement with our results, Levine and coworkers [11] showed that patients following a 24 week walking program have lower depressive scores and were abstinent from binges. Another study [35] demonstrated that a 4 month program combining CBT with aerobic exercise results in fewer depressive symptoms and fewer binges per week than CBT alone.

### 4.3. Effects of CAAET on Exercise Capacity

Regarding exercise capacity, we observed an improvement in both groups after 6 months of training. However, only CAAET women significantly increased the distance covered during 6MWT. These results mean that the two interventions improved exercise capacity, but the CAAET intervention was more efficient in improving the aerobic aspect of physical performance (*d* 6MWT: 1 vs. 0.6, CAAET and CTRL, respectively). Interestingly, we did not find the same result for muscle strength even if the effect sizes of differences between PRE and POST in CAAET compared to the CTRL group showed a greater improvement for the women who followed the physical activity program (*d* ST: 0.7 vs. 0.3, CAAET and CTRL, respectively). This may be because the physical activity program was focused principally on brisk walking (60 min), which can be considered the most natural and easiest activity to learn and perform, and only partly on strength exercises (20 min). This aspect could justify the significant improvement obtained only in aerobic capacity in CAAET women. In addition, BED patients had a lower level of weekly physical activity participation and a lower physical self-perception, which could be associated with a lower functional exercise capacity; women may refuse to undertake physical activity due to a number of barriers, such as social physique anxiety, health problems, reduced level of fitness, lack of social support, and limited access to facilities [36]. Overcoming these barriers is a first step toward promoting participation in exercise training interventions.

Another feature of obese people is a relative reduction in skeletal muscle strength and reduced cardiorespiratory fitness [37]. As a consequence of aerobic training, resting sympathetic activity is reduced and vagal tone is increased, with potential effects on blood pressure, thrombosis, and other factors associated with coronary risk [38]. Low levels of cardiorespiratory fitness, as expressed by low peak pulmonary oxygen uptake (VO_2_), are associated with an increased risk of cardiovascular morbidity and mortality [39,40]. Strength training is able to generate peripheral adaptations, leading to hypertrophy and other physiological adaptations [41,42] that are different to those involved in aerobic training in terms of central hemodynamics stimulation [43]. Indeed, the limited heart rate and cardiac output responses are counterbalanced by high stimulation of the peripheral circulation during small muscle mass exercise [44].

Interestingly, aerobic training may lead to some gains in maximum strength [45], and strength training may also improve aerobic capacity [46], showing that some synergistic benefits of concurrent aerobic and strength training may occur. Therefore, multiple mechanisms exist by which the exercise training may provide benefits.

## 5. Conclusions

The strength of our study is a comprehensive assessment of BED-related symptoms, such as the objective assessment of physical activity parameters using specific motor tests, including of the control group. To date, a number of studies have used only self-reported questionnaires and structured diagnostic interviews to assess physical activity levels [47].

The major limitation of this study is the short duration of CAAET. It is of interest, however, that a short duration of CAAET is sufficient to improve anthropometric parameters, eating disorder symptoms, and exercise conditions. In addition, it is important to remember that all of these parameters are relevant to the occurrence of eating disorders. Higher-powered confirmatory physical activity programs are necessary to examine different types of exercise and different doses of physical activity at different time points in the BED experience to obtain a full assessment of the effects of physical activity that are important for these women. In addition, future studies are needed to capture the same features in a large sample size, with more diversity in age range and inclusion of both women and men.

Our findings suggest that both interventions similarly improved BED symptoms. The addition of CAAET provides no additional benefit in improving the effects of the dietary and CBT program. However, it could be important in the long-term maintenance of both body weight loss and reduction in binge eating episodes in BED patients, thus improving the eating disorder.

## Figures and Tables

**Table 1:** Anthropometric characteristics of women with binge eating disorder (BED).

Mean ±SD	PRE		POST	
	CTRL (*n* = 9)	CAAET (*n* = 10)	CTRL (*n* = 9)	CAAET (*n* = 10)
Weight (kg)	107 ± 32	101 ± 21	102 ± 28 *	87 ± 14 *
BMI (kg/m^2^)	38 ± 10	38 ± 6	36 ± 9 *	32 ± 3 *

CTRL, control group; CAAET, Combined Aerobic and Anaerobic Exercise Training (intervention group). Mean (±SD) values of weight (kg) and body mass index (BMI) (kg/m^2^) in women with BED, before (PRE) and after (POST) 6 months of Combined Aerobic and Anaerobic Exercise Training. * *p* < 0.05 PRE vs. POST.

**Table 2:** Eating disorder symptoms evaluation of women with BED.

Mean ±SD	PRE		POST	
	CTRL (*n* = 9)	CAAET (*n* = 10)	CTRL (*n* = 9)	CAAET (*n* = 10)
BES (score)	23 ± 9	23 ± 10	15 ± 7 *	10 ± 8 *
BITE Symptom (score)	14 ± 6	15 ± 7	9 ± 3 *	7 ± 4 *
BITE Severity (score)	9 ± 7	8 ± 7	6 ± 4 *	3 ± 3 *

CTRL, control group; CAAET, Combined Aerobic and Anaerobic Exercise Training (intervention group). Mean (±SD) values for Binge Eating Scale (BES) (score) and Bulimic Investigation Test Edinburgh (BITE) (score) in women with BED, before (PRE) and after (POST) 6 months of Combined Aerobic and Anaerobic Exercise Training. * *p* < 0.05 PRE vs. POST.

**Table 3:** Exercise capacity of women with BED.

Mean ±SD	PRE		POST	
	CTRL (*n* = 9)	CAAET (*n* = 10)	CTRL (*n* = 9)	CAAET (*n* = 10)
6 MWT (m)	450 ± 112	507 ± 74	520 ± 112 *	612 ± 90 *^,^°
ST (n°/30 s)	19 ± 6	22 ± 6	21 ± 7 *	26 ± 5 *

CTRL, control group; CAAET, Combined Aerobic and Anaerobic Exercise Training (intervention group). Mean (±SD) values for Six-Minute Walk Test (6 MWT) (m) and Squat Test (ST) (n°/30 s) in women with BED, before (PRE) and after (POST) 6 months of Combined Aerobic and Anaerobic Exercise Training. * *p* < 0.05 vs. PRE, ° *p* < 0.05 CAAET vs. CTRL.
